# Editorial: Microbiome-based metabolomics revolution in health and microbial metabolites redefinition

**DOI:** 10.3389/fcimb.2025.1582602

**Published:** 2025-04-09

**Authors:** Raja Ganesan, Durairaj Thirumugan

**Affiliations:** Department of Biotechnology, Faculty of Science and Humanities, SRM Institute of Science and Technology, Chennai, Tamil Nadu, India

**Keywords:** microbiome, metabolomics, metabolites, biomedical applications, human health and diseases, hepatology

The Frontiers in Cellular and Infection Microbiology Research Topic “Microbiome-Based Metabolomics Revolution in Health and Microbial Metabolites Redefinition” highlights state-of-the-art research to understand microbial metabolisms, metabolites, and metabolic regulation. This Research Topic covers many facets of microbial metabolomics research, ranging from basic biological principles to microbial metabolites and their metabolic redefinitions.

## Healthy individuals reference microbiome profiles

Reference microbiome profiles have potential diagnostic potential, but further studies involving larger, diverse cohorts are needed to validate their biological and clinical significance. Extending the approach to populations of different races, ethnicities, and geographic regions is crucial for generalizability. Understanding the influence of genetic predispositions, dietary habits, and environmental exposures on microbiome composition is essential for global reference intervals. Lifestyle factors, such as BMI, alcohol consumption, and tobacco smoking, should be considered. The FC approach, which employs advanced statistical models, has limitations, especially for low-abundance taxa. Validating these profiles through complementary methods could improve their accuracy and reliability (Oh and Park).

## Vitamin D interacts with gut microbiota but not anandamide: a preliminary study

The vitamin D intake can significantly influence gut microbiota composition. Deficiency in vitamin D can lead to dysbiosis and gastrointestinal inflammation. Studies show that supplementation can increase gut microbial diversity and the ratio of *Bacteroidetes* to *Firmicutes*. Vitamin D intake is linked to a higher abundance of certain bacteria while reducing the abundance of others. In women with pre-eclampsia, a negative correlation was found between Vitamin D levels and the relative abundance of Dorea. Dorea plays a crucial role in maintaining gut barrier integrity and preventing pre-eclampsia. Vitamin D receptors in intestinal enterocytes facilitate the production of antimicrobial peptides, promoting anti-inflammatory responses and reducing infection risk. Overall, vitamin D status can potentially prevent pre-eclampsia by maintaining the gut microbiome (Han et al.).

## Gut-liver axis microbiome interactions and liver cancer pathogenesis

This review emphasizes the role of the gut-liver axis in liver disease pathogenesis, particularly HCC. It highlights the importance of microbial metabolites, gut barrier integrity, and immune modulation in driving inflammation and carcinogenesis. The review addresses controversies and suggests future studies, humanized models, and standardized microbial compositions for personalized treatment. It also calls for multicentre clinical trials to validate microbiota-targeted interventions (Wang et al.).

## Comparative genomic analysis

In summary, *Klebsiella pneumoniae* infections are known to be caused by epidemiological risk factors, including food, livestock, companion animals, environmental factors, and a location’s demographics. According to the current study, food may serve as a possible harbour for harmful *Klebsiella pneumoniae*. This is the first study to reveal fish isolates of the unusual serotype O3b *Klebsiella pneumoniae* as well as several STs, including ST37, ST515, and ST192 (Krishnan et al.).

## Female cervical dysplastic: vaginal metabolites and microbiota

The study focuses on the vaginal microbiota or metabolites’ potential for early cervical cancer detection. The combination of vaginal metabolites and vaginal microbiota may be a more accurate predictor of cervical cancer, according to a linear regression model. The diagnostic performance was higher when vaginal metabolites and vaginal microbiota were combined. Since the incidence of cervical cancer is rising, early detection is essential (Yu et al.).

## The biomedical applications of marine fungi-derived secondary metabolites: FGFC

Fungi-derived compounds which may be isolated from various marine fungi and have a range of biological activity, are reviewed along with their possible medicinal uses. Fungi fibrinolytic compounds may treat thrombolysis, renal damage, bleeding, neurological degenerative diseases, and inflammatory responses. However, this includes the need for additional studies on other significant issues and the paucity of studies on innovative biomaterial platforms for effective drug administration (Jeevithan et al.).

## The gut microbiota can reduce anxiety through intestinal aminobutyric acid

According to the study, TMC3115 and LGG enhance brain-derived neurotrophic factor expression, which in turn promotes neuron function and development. The impacts on behavioral traits, however, were only seen in mice given TMC3115 and not LGG. This implies that modifications in the gut microbiota, particularly the growth of GABA-producing bacteria, may be the mechanism through which TMC3115 exerts its effects. According to the study, TMC3115 treatment may influence intestinal GABA and IEC function together, which could have a coordinated effect on gut-brain equilibrium (Ikegami et al.).

## Postpartum depression: gut microbiota and blood metabolites

Using metagenomic sequencing techniques, this study investigates the causal link between blood metabolites, genetically modified (GM) foods, and postpartum depression (PPD). GWAS data mostly including European populations, a lax threshold, and limited applicability to diverse ethnic groups are some of the research’s drawbacks. Additionally, the study did not include age-related stratification and used GM data from both male and female patients, eliminating sex chromosomes (Cui et al.). The results point to possible therapy approaches and provide a theoretical framework for locating biomarkers linked to PPD.

## Translational research in liver diseases based on microbiome-derived metabolic functions

Changes in gut microbial composition are associated with chronic liver illness, and ethanol/alcohol-induced liver disease can be passed on by fecal microbiota transplantation. The development of metabolic-related liver disease is influenced by the leaky intestinal barrier, which results in fibrotic response, hepatocyte loss, and inflammation. Changes in the immune system, bile acid composition, and gut microbial activity are all associated with gut dysbiosis. In metabolic-related liver illness, the gut microbiota is impacted by non-targeted therapeutic methods such as FMT, probiotics, antibiotics, and various dietary modifications.

In conclusion, the application of microbiome and metabolomics methodologies has been significantly transformed to investigate the role of the microbiome in human diseases. The microbiome plays a crucial role in maintaining overall health by aiding the immune system and protecting against metabolic disorders. The gut microbiome also helps in regulating metabolic diseases. The balanced microbiome is associated with reduced fatty liver risk, and liver cancer metabolisms.

The challenge in studying microbiome-related metabolites in this Research Topic is the complexity and diversity of microbial communities in diverse disease patterns and healthy individuals. Moreover, the dynamic nature of various disease patterns makes it difficult to pinpoint specific metabolic effects on health. Furthermore, the metabolic interactions between the host and metabolic diseases can vary significantly, complicating the interpretation of their metabolic impact.

